# A sustainable acoustic customization of open porous materials using recycled plastics

**DOI:** 10.1038/s41598-022-14009-z

**Published:** 2022-06-29

**Authors:** Marco Caniato, Luca Cozzarini, Chiara Schmid, Andrea Gasparella

**Affiliations:** 1 Faculty of Science and Technology, Free University of Bozen, 39100 Bolzano, Italy; 2 Department of Engineering and Architecture, University of Trieste, 34127 Trieste, Italy

**Keywords:** Engineering, Materials science

## Abstract

Foams are commonly used as sound absorbers and thermal insulators for many industrial and construction applications. The insulating materials market is currently dominated by inorganic fibres like glass and mineral wool, as well as plastic foams. However, worldwide plastics consumption produces huge amounts of waste, generating concerns about soil, air and especially seawater pollution. Hence, new methods for recycling marine microplastic litter according to cleaner production criteria are being sought. This paper presents a novel, sustainable and eco-friendly foamy material made of microplastic waste, namely polyethylene terephthalate (PET) and polystyrene (PS), incorporated into a bio-based matrix. Samples with different compositions were prepared and then characterized for sound absorption properties. Evidence is presented of very good acoustic performances and of how the acoustic characteristics of the end product can be customized using different microplastic content and type. This allows envisioning many industrial and civil applications for this novel open-cell material.

## Introduction

Foams are commonly used as sound absorbers and thermal insulators in everyday life, industrial and building applications^1^. They can be found in many industrial and construction applications. For example, in the automotive industry, it is common to incorporate them in car doors, dashboards, bases etc.^2–4^. In construction, these insulators are used within double-leaf walls^5^, façades^6^, windows^7^ and for many other purposes^8^. In buildings, foams are used as thermal insulators and as sound absorbers. Foams used in indoor environments can provide noise control, reducing reverberation in rooms or working spaces^9^. For similar reasons they are also widely used in the naval industry^10–12^.

In such applications, their sound absorption is of paramount importance and today their overall performance can be customized. New tuneable materials developed with enhanced acoustic performance in mind are now a superior alternative to conventional foams^13,14^. However, the insulating materials market is currently dominated by inorganic fibrous materials (glass and mineral wool) and organic foamy ones (expanded or extruded polystyrene and polyurethane)^15,16^. These conventional insulators are produced from primary raw materials, such as rocks and fossil fuels. The use of secondary or renewable sources for their production is nowadays being evaluated to comply with sustainability and ecological requirements^15,17^.

The benefits of such novel materials in terms of environmental indices and life cycle analysis are evident^18^. High plastic consumption worldwide is generating huge amounts of waste, year after year. Macro and micro plastic litter affect habitats everywhere, especially marine environments^19^. Unfortunately, plastic is particularly difficult to retrieve from the sea, since it tends to break up into smaller pieces due to wind, water movement and solar irradiation^20–23^. Hence, its end-of-life handling and management has become a major issue. Nevertheless, it is common to burn plastic wastes^24,25^, or to use them as fillers for something completely different from their original purpose^26,27^. Although many lines of research are focusing on innovative means of recycling plastics, new recycling methods for marine plastic litter, pursuing cleaner production criteria, are urgently required.

Accordingly, this article presents a sustainable foam produced using a bio-based matrix incorporating waste plastic powder. The natural polymer selected as its bio-based matrix is alginate, a linear anionic polysaccharide extracted from brown algae that is already widely known for its suitability as biocompatible scaffold^28,29^. It is therefore possible to produce porous structures with very good acoustic properties using this natural low cost material^30,31^. Thus, by combining the bio-bases matrix with the microplastic powder, it was possible to develop an innovative open-cell foam.

Previous research^32^ has shown that it is possible to produce foams with interesting thermal and acoustic properties with medium-density alginate. Other elements such as plasticizers may be added to the bio-matrix in order to achieve some other properties, like resilience. Among plasticizers, in the case of alginate foam, triol-like components are already in use as a possible option in similar contexts^33–35^.

To the best of the authors’ knowledge, low-temperature, blowing agent-free foaming methods are not currently available. Thus, this sustainable foam produced using alginate matrix and plastic waste powder represents a novel, cleaner, open-cell material, whose thermal and acoustic properties may be desirable in many fields.

Therefore, the main aim of this research is to produce and test a sustainable foam made from recycled microplastic, namely polyethylene terephthalate (PET) and polystyrene (PS). The samples are produced in order to be tuneable for sound absorption purposes.

A previous work, devised by one of the authors, performed an LCA assessment, based on the same patent-pending procedure^18^. The benefits of this novel material in terms of environmental indexes are here highlighted as well as its sustainability.

## Materials and methods

### Microplastics selection

Plastics from industrial and domestic waste, namely polyethylene terephthalate (PET) from bottles and rigid foams as well as rigid and expanded polystyrene (PS), were chosen as representative of the macro and micro plastic waste most commonly found in the marine environment^36–38^. Polyethylene terephthalate powders were obtained by further grinding rigid PET foam, polystyrene powders by grinding rigid PS and PET flakes by grinding PET bottles.

After grinding, plastic particles were sieved in two successive stages, using a 5 mm and 2 mm mesh sieves. After these steps, all the particle dimensions were less than 5 mm, meaning that they could be defined as “microplastics” according to the approved international standard^38^.

### Production methods

Foam production was carried out via a sol–gel process, according to a procedure, pending patent, devised by one of the authors and previously reported^32^. Alginic acid sodium salt from brown algae (alginate, medium viscosity), glycerol (≥ 99.5%), D-gluconic acid δ-lactone (GDL, ≥ 99.0%) and calcium carbonate (CaCO_3_, 98%) necessary for the sol–gel process were purchased from Sigma Aldrich. Briefly, the alginate was mixed with water, plastics (see Table 1 for composition details), CaCO_3_ and GDL; the mixture was then poured into Petri dishes (used as sample holders).

**Table 1:** Details of the foam samples’ composition.

Sample ID	Alginate	Glycerol	Total microplastics	PET powder	PET flakes	PS powder
	[w/v%]	[mL]	[g]	[g]	[g]	[g]
1	1.4	0.0	3.0	3.0	0.0	0.0
2	1.6	0.0	3.0	3.0	0.0	0.0
3	1.8	0.0	3.0	3.0	0.0	0.0
4	2.0	0.0	3.0	3.0	0.0	0.0
5	1.4	0.8	3.0	3.0	0.0	0.0
6	1.4	1.6	3.0	3.0	0.0	0.0
7	1.4	3.2	3.0	3.0	0.0	0.0
8	1.4	0.0	3.0	0.0	0.0	3.0
9	1.4	0.0	3.0	0.5	0.0	2.5
10	1.4	0.0	3.0	1.0	0.0	2.0
11	1.4	0.0	3.0	2.0	0.0	1.0
12	1.4	0.0	3.0	2.5	0.0	0.5
13	1.4	0.0	3.0	2.0	1.0	0.0
14	1.4	0.0	3.0	1.5	1.5	0.0
15	1.4	0.0	3.0	1.0	2.0	0.0
16	1.4	0.0	3.0	0.5	2.5	0.0

The viscosity of the alginate—microplastics suspension provides homogeneous dispersion through stirring. Accordingly, the alginate concentration was experimentally optimized in the range 1.4–2.0% w/v.

A three-dimensional porous hydrogel network is formed after the Ca^+2^ ions, slowly released from CaCO_3_, crosslink with the G-blocks of the polysaccharide; the pH of the solution gradually decreases due to GDL hydrolysis in water. The principle of this synthesis route is based on the initial formation of a three-dimensional porous hydrogel network. This structure is then preserved by freeze-drying, a process that eliminates liquid water and prevents the consequent collapse of pores^39^. The water entrapped during gelation is then removed, leaving a porous structure. Three samples were produced for each composition to assess the consistency of the results obtained from different samples of the same material.

### Production customization

The morphology of the foam and therefore its acoustic performance depends on various factors, which can be broadly reduced to (i) type of matrix and (ii) type of filler. These aspects are discussed in more detail in the following paragraphs. Using this production method, it is possible to obtain samples of various dimensions and thicknesses. In this case, samples were produced circular, featuring a diameter of 4.5 cm, according to the performed tests. Intentionally, the microplastic powder was accumulated on just one side of the samples, in order to produce a significant difference in density.

### Matrix variation

As regards the matrix, the most influential factors are the concentration of gelling agent (alginate) and the presence or absence of a plasticizer. According to a recipe previously reported^35^, the plasticizer used in this case was glycerol. An initial set of samples was prepared with a fixed type and concentration of filler, without glycerol but with varying concentrations of alginate, between 1.4 and 2.0 w/v %, to test the influence on the properties of the resulting foam (samples 1–4 in Table 1). Another set of samples was made with fixed alginate (1.4 w/v %) and filler contents (3.0 g PET powder), but with increasing glycerol contents (samples 5–7 in Table 1).

### Powder and flakes variation

Regarding the type of particles, the influential factors could be the amount of plastics, as well as their shape/size and chemistry. As mentioned previously, the latter was restricted to PET and PS, while two shapes/sizes were chosen: powder (PS mean size 0.88 ± 0.53 µm; PET mean size 0.85 ± 0.36 µm) and flakes (PET mean size 1.37 ± 0.45 µm). It was decided to keep the matrix composition fixed, with sample 1 (Table 1) considered as a reference.

The total plastic weight was kept constant (3.0 g), but different microplastics compositions were tested. Specifically, ratios of PET powder to PS powder on the one hand and PET powder to PET flakes on the other were varied to simulate mixed microplastics and to test their respective influence on the properties of the resulting foam (samples 8–16 in Table 1).

### Acoustics test

**Figure 1:**
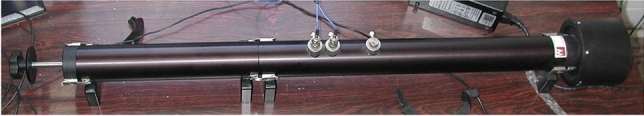
Kundt tube.

A 2-microphone plane wave impedance tube (Fig. 1) was used to determine the samples’ sound absorption properties, according to the EN ISO 10534-2 standard^40^. This procedure was adopted to investigate surface impedance and complex reflection index and three samples were tested for every measurement.

By the use of the transfer functions between microphones it is possible to measure the normal incidence surface impedance and sound absorption coefficient.

The experimental set-up consists of:

- Plane-wave tube having a diameter of 45 mm;
- 2 ¼″ prepolarized microphones PCB 377C10 (mounted at a distance of 30 mm);
- Power amplifier B&K type 2716C;
- NI USB 4431 for generation and acquisition of test signals;
- Acquisition ad post-processing software developed on Labview® platform.

Measurement set-up is calibrated by using a crossed-microphones procedure for compensating amplitude and phase mismatch of transducers and acquisition device as described in^40^.

The pressure of the incident and reflected wave at the points of the tube are expressed by Eqs. (1) and (2):

1 $${p}_{i}(x)= {\widehat{p}}_{i}{e}^{i{k}_{0}x}$$

2 $${p}_{r}(x)= {\widehat{p}}_{r}{e}^{-i{k}_{0}x}$$

where *p*_*i*_ is the incident pressure, *p*_*r*_ is the reflected pressure, *x* represents the mono-dimensional space, ${\widehat{p}}_{i}$ and ${\widehat{p}}_{r}$ represent the amplitude of the incident and reflected sound wave respectively, and *k*_*0*_ is the wave number.

The sound pressure at microphone positions x_1_ and x_2_ can be measured as expressed via the following Eqs. (3) and (4):

3 $${p}_{1}(x)= {\widehat{p}}_{i}{e}^{i{k}_{0}{x}_{1}}+{\widehat{p}}_{r}{e}^{-i{k}_{0}{x}_{1}}$$

4 $${p}_{2}(x)= {{\widehat{p}}_{i}{e}^{i{k}_{0}{x}_{2}}+\widehat{p}}_{r}{e}^{-i{k}_{0}{x}_{2}}$$

Using the transfer matrix method, it is then possible to obtain the complex reflection index *R*_*p*_, which can be used to compute the sound absorption coefficient (Eq. (5)) as follows:

5 $$\alpha = 1- {\left|{R}_{p}\right|}^{2}$$

In order to assess which parameter most affects sound absorption frequency trends, a Johnson-Champoux-Allard (JCA) model was used^41,42^. The five parameters were obtained by a regressive procedure featuring minimization of the results^43^. A nonlinear best-fit (NLBF) was used featuring the Nelder-Mead Simplex Theory^44^. No numerical or analytic gradients are used in this procedure as it finds the minimum related to a scalar function considering disparate parameters, using an initial constrained value. Thus it is commonly attributed to a constrained nonlinear optimisation, as it is possible for the algorithm to use the imposed limits for all considered variables. Thus, a minimum and a maximum values were provided.

This model is based on the microstructure determination by means of porosity, tortuosity, airflow resistivity, thermal characteristic length and viscous characteristic length. The JCA model was successfully used for open-cell foams microstructure in many studies available in the literature^45–48^.

**Figure 2:**
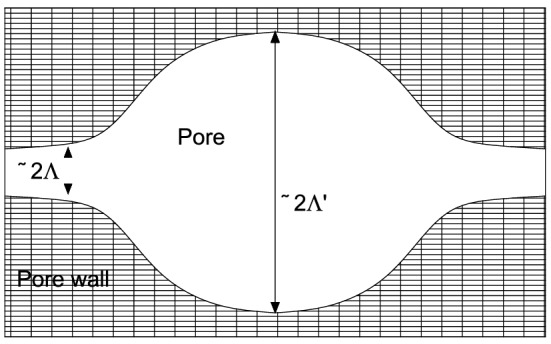
Schematic representation of thermal characteristic lengths.

From a modelling point of view, porosity (ϕ) tortuosity (α_∞_) airflow resistivity (σ), thermal characteristic length and viscous characteristic length (Λ, Λ’) represent the five most important parameters to be used to model an open microstructure of a material. Porosity represents the ratio between the effective material presence and the contained air; tortuosity speaks for the free mean path crossed by the sound wave throughout the material thickness; airflow resistivity expresses the resistance opposed by the microstructure to let an airflow pass through it. The thermal characteristics lengths are geometrical properties represented in Fig. 2.

Literature used this approach in time and many papers were published in this last decade. As an example, Bonfiglio and Pompoli^49^ in their review collected the main papers on this topic, demonstrating that both from surface impedance and from sound absorption coefficient. Both parameters were measured in this work and used in the regressive procedure.

### Microstructure analysis

Computed microtomography using X-ray (X-µCT) was achieved by means of a micro-focused X-ray source which conveyed a polychromatic (40–130 kV), featuring a minimum focal spot size of 5 μm. The obtained 3d microstructure was then analysed using FIJI software featuring Bone J retrieving the porosity of some materials.

In order to provide a more wide characterization, the airflow resistivity of some layers was investigated. Airflow resistivity is a measure of the resistance that air flow meets passing through a structure. The equipment and procedure for the determination of this parameter is described in the International Standard ISO 9053-2^50^. The alternating method is used, and a low frequency (2 Hz) airflow is generated by means of a rigid piston within a measurement chamber. By using a microphone, the pressure drop across the tested material is measured and the airflow resistivity is determined as follows:

6 $$\upsigma =\frac{{\mathrm{P}}_{\mathrm{eff}}}{\mathrm{v}\cdot \mathrm{d}} \, [\text{Ns/}{\text{m}}^{4}]$$

being v [m/s] the airflow velocity and d [m] the thickness of the specimen. Tests are carried out at different airflow velocities (0.43 mm/s, 1.07 mm/s, 2.14 mm/s and 4.02 mm/s) and by means of a linear regression with zero intercept the pressure drop is estimated at the reference value of the airflow velocity (0.5 mm/s).

**Figure 3:**
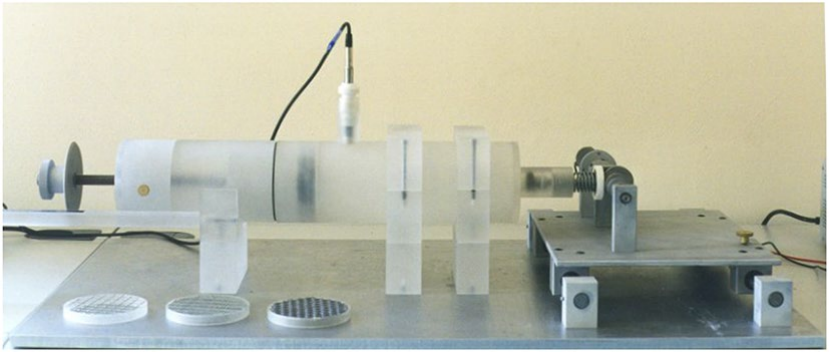
Experimental test-rig used for airflow resistivity measurements.

The measurement lay-out includes a Bruel & Kjaer Type 4165 condenser microphone with Larson-Davis model 900B preamplifier and a National Instruments NI 4431 for signal acquisition and post-processing. Figure 3 shows the experimental equipment used for airflow resistivity measurements.

## Results and discussion

### Production results

**Figure 4:**
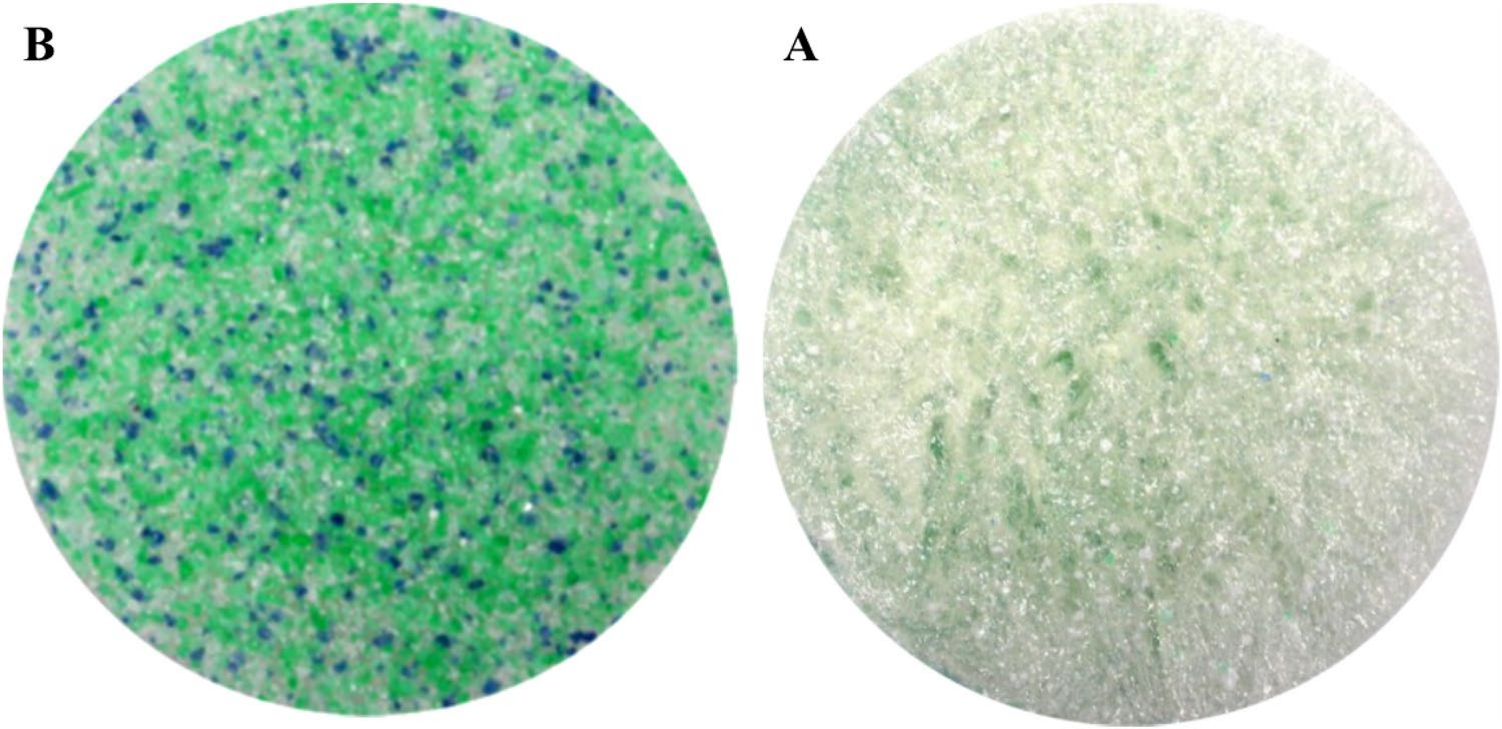
Images of sample 13, showing PET flakes at the bottom (left side, **B**) and PET and/or PS powders at the top of the sample (right side, **A**).

As prepared in Petri dishes (4.5 cm diameter), the resulting foams presented two visually different sides. The lower one, in contact with the dish (identified with the letter B, bottom), appears smoother, while the upper side, in contact with the air (identified with the letter A, air), is rougher. An example is shown in Fig. 4. From these images, it is also possible to see how the PET flakes tend to sink to the bottom of the Petri dish (they are clearly visible on side B), while the PET and/or PS powders remain suspended and are therefore more homogeneously dispersed in the matrix.

**Figure 5:**
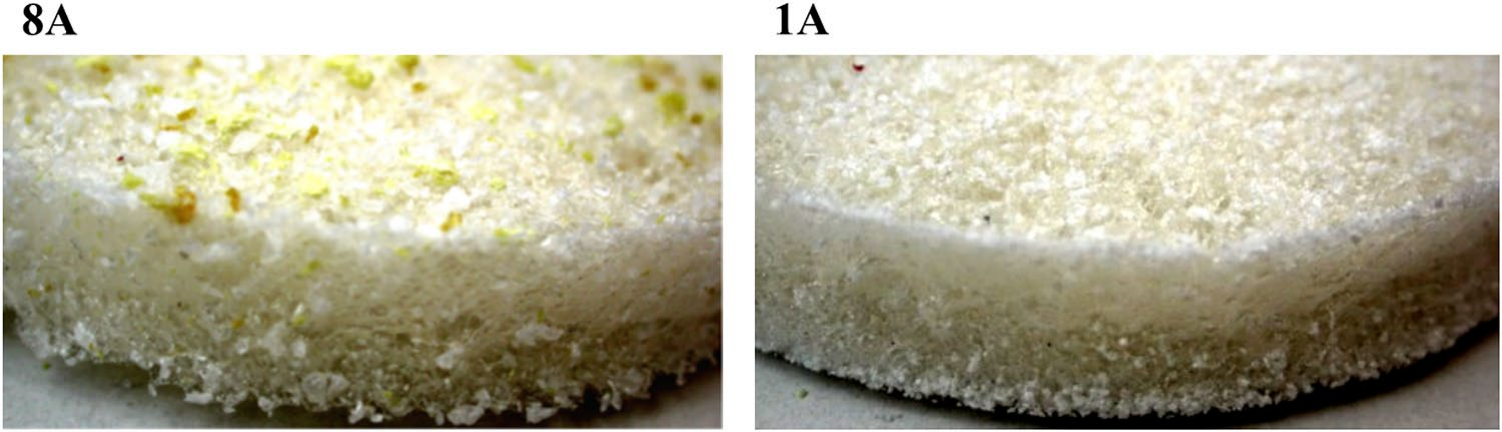
Pictures of upper sides of samples 8 (PS powder, left) and 1 (PET powder, right).

The type and density of plastic also influences the dispersion. Figure 5 shows an example of two samples both loaded with powder, only PET (d = 1.38 g/cm^3^) in the first case (sample 1), and only PS (d = 1.05 g/cm^3^) in the second (sample 8). As the images show, PET tends to accumulate on the bottom of the Petri dish, while PS tends to remain on the surface. Then, we use this property to intentionally accumulate microplastics on just one side of the samples.

**Figure 6:**
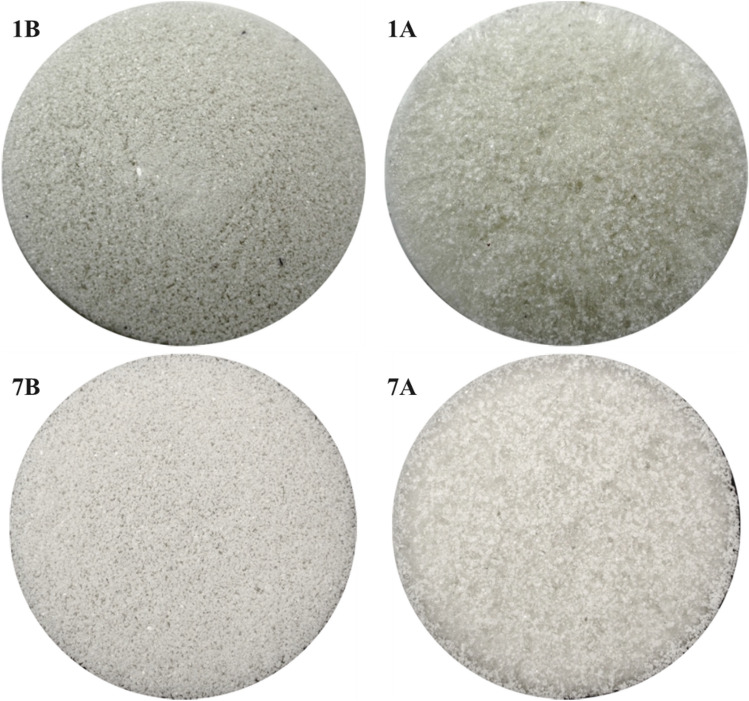
Pictures of sides B and A of sample 1 (without glycerol) and sample 7 (3.2 ml glycerol).

The concentration of glycerol contributed to a more homogeneous stratification of PET powders, as can be seen in Fig. 6, where sides A and B (upper and lower, respectively) of sample 1 (without glycerol) are compared to those of sample 7 (highest concentration of glycerol).

### Microstructure analysis

**Figure 7:**
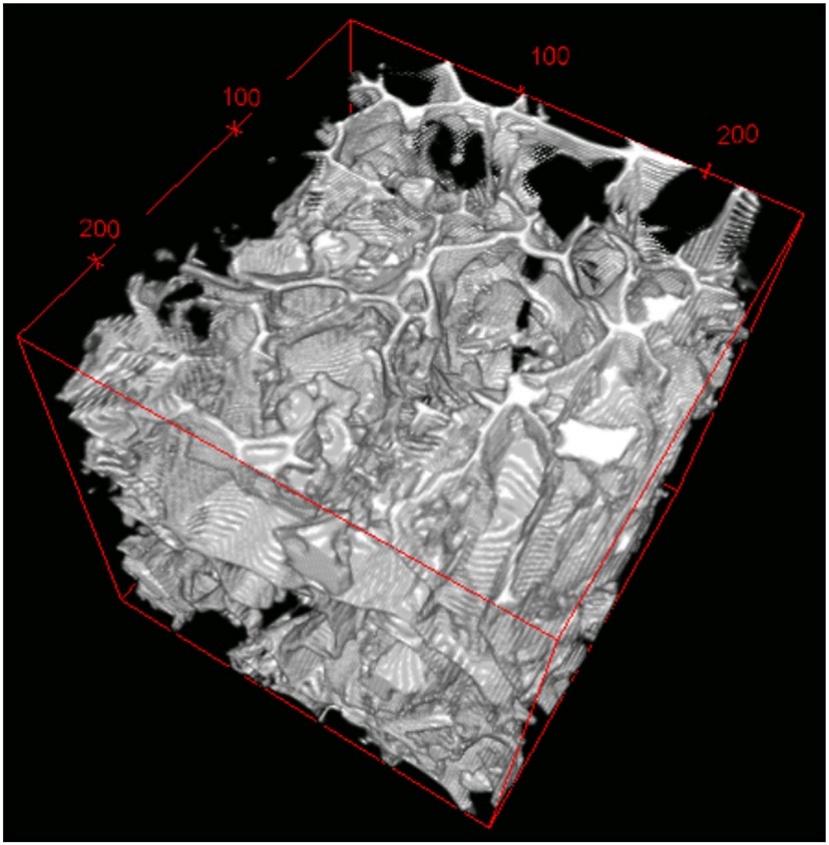
The 3D image of the foam microstructure (Volume of Interest of 250 μm × 250 μm × 250 μm).

In Fig. 7, a micro-cT 3D image is reported, evidencing how the foam features an open-pore structure, obtained using Fiji software.

Using Bone J, some validation of the JCA parameters can be performed. Even if the validation of this procedure is available in the literature, for two samples (sample 1 and sample 2) a comparison is provided in Table 2.

**Table 2:** Validation of the process—porosity.

Sample ID	Measured Porosity [–]	Retrieved Porosity [–]
1	0.76	0.75
2	0.73	0.73

In order to provide a more extended validation, airflow resistivity of some materials was tested, compared to the retrieved one and compared.

Interestingly, the differences reported in Tables 2 and 3 are in accordance with literature^49^.

**Table 3:** Validation of the process—airflow resistivity.

Sample ID	Measured airflow resistivity [Ns/m^4^]	Retrieved airflow resistivity [Ns/m^4^]
1	1663	1827
2	1114	1158
8	5668	5489
12	3305	3479

### Acoustics results

The impedance tube measurements revealed several sound absorption trends. In the following section, the respective influence of varying matrix, powder and flakes content are analysed and discussed.

#### Matrix variation results

**Figure 8:**
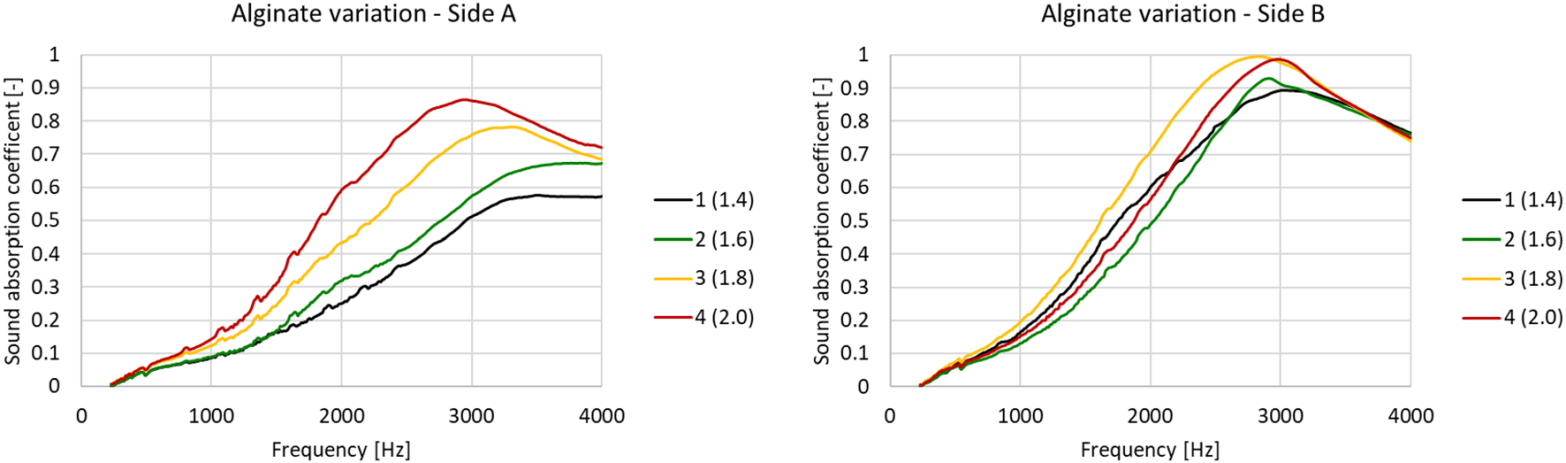
Sound absorption coefficient frequency variation at increasing alginate concentrations.

The measured sound absorption coefficient trends as a function of alginate concentration are reported in Fig. 8.

Clearly, when focusing on side A, the alginate content modifies the sound absorption frequency trend. Specifically, the more alginate is used, the more the performance is improved. At 1.8 w/v %, we can also observe a modification of the sound absorption peak. Using a minimization approach, the microstructure parameters are reported in Table 4.

**Table 4:** Alginate variation—JCA model parameters.

Sample ID	Alginate conc. [w/v %]	Airflow resistivity [Ns/m^4^]	Porosity [–]	Tortuosity [–]	Viscous characteristic length [μm]	Thermal characteristic length [μm]
1	1.40	1663	0.75	1.01	191	287
2	1.60	1114	0.73	1.01	132	273
3	1.80	5264	0.74	1.37	154	280
4	2.00	2020	0.73	1.55	124	258

It is evident that the internal structure of the foam does not change significantly with increasing concentrations of alginate. Accordingly, the thermal characteristic lengths present similar dimensions and airflow resistivity remains substantially unchanged, except in the third sample (1.8 w/v%). Furthermore, porosity stays constant, while the viscous characteristic length decreases. However, tortuosity appears to increase as alginate concentration increases. This implies that the free mean path of the sound waves increases (tortuosity increasing) and that the connections between cells decrease, thereby hampering the exchange of air between the different micro-volumes. As an overall result, on side A, the sound absorption coefficient rises to its maximum at about 3400 Hz–3500 Hz. When more alginate is added, the result is to move the absorption peak to lower frequencies (about 2700 Hz). Sample 3 presents a higher airflow resistivity, compared to samples 1, 2 and 4. This is due to the fact that at that precise alginate concentration, the microplastics tend to accumulate more on the surface A, thus opposing more resistance to the airflow.

It is interesting to note that the range 1000 Hz–3000 Hz also benefited from alginate addition. As shown in Table 5, where α_w_ is the averaged sound absorption coefficient computed in the range 1000 Hz–4000 Hz, starting from the first case (1.4 w/v%) we find an overall increase in sound absorption.

**Table 5:** Averaged sound absorption coefficient at increasing alginate concentrations.

Sample	Alginate conc. [w/v%]	α_w_—side A	α_w_—side B
1	1.40	0.37	0.59
2	1.60	0.47	0.57
3	1.80	0.55	0.65
4	2.00	0.61	0.62

It is clear from these results that increasing the alginate concentration (thereby reducing the dimensions of the internal pores) and prolonging the free mean wave path cause an overall improvement in performance. Global properties improvement was 24%, 10% at the first step (1.4–1.6 w/v%), 8% at the second step (1.6–1.8 w/v%), and 6% at the final step (1.8–2.0 w/v%).

When focusing on side B, a different picture emerged. The significantly higher surface impedance exposed to the plane noise wave acts primarily as an obstacle, i.e., a resistive layer. As such, it is of no particular interest to study its microstructure. Side B of all samples provided better performance than their respective side A and increasing the alginate had little to no effect. In fact, only by comparing the first two alginate concentrations (1.4 and 1.6 w/v%) to the last two (1.8 and 2.0 w/v%) we find a positive influence due to the microstructural changes. Specifically, the latter two samples provided better performance with respect to the former two, which displayed similar frequency trends. The averaged values (Table 5) confirm that sample 3 (1.8 w/v%) and sample 4 (2.0 w/v%) exhibited better performance, while there was little difference between the other two. Therefore, it appears that sound absorption properties are significantly enhanced by the addition of a high impedance surface on just one side of the material; when this side is exposed to noise there is no considerable influence of microstructure modification, while this is a significant factor for the other side, without a high-impedance surface.

Overall, the addition of alginate to the foam improved acoustic absorption at all tested concentrations when considering both side A and side B (resistive layer).

**Figure 9:**
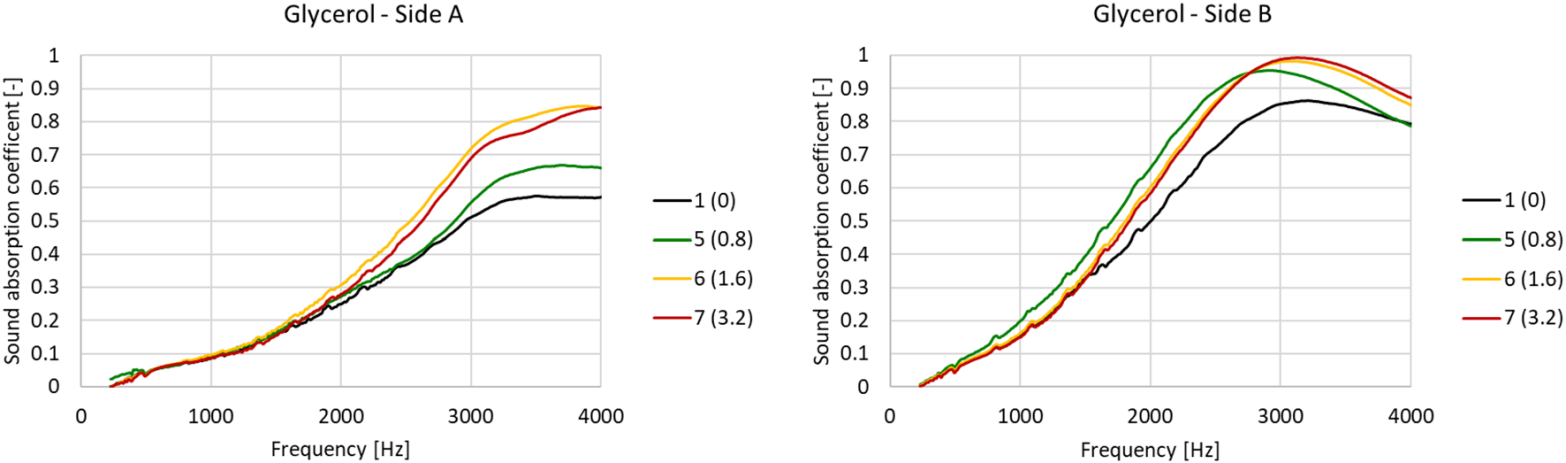
Variation in the sound absorption frequency coefficient at increasing glycerol concentrations.

Similarly, Fig. 9 shows the measured frequency trends measured for increasing concentrations of glycerol. Focusing on side A, the effect was similar to the one of adding alginate, albeit to a lesser extent. Specifically, the sound absorption increased, starting from a frequency of about 2000 Hz, when adding glycerol.

Table 6 reports the five Johnson-Champoux-Allard (JCA) parameters, calculated using a minimization approach. Even if generally a decrease is assessed, the glycerol concentration increase did not change airflow resistivity and tortuosity significantly, as for all the other parameters. Porosity decreased incrementally with increasing concentrations of glycerol, albeit to a lesser extent than viscous characteristic length, which displayed significant reductions as glycerol concentration increased. This means that the microstructure is affected by glycerol, which decreases porosity and cell size, while increasing cell wall thickness and influencing overall mechanical performance.

**Table 6:** Glycerol concentration variation—JCA model parameters.

Sample	Glycerol [mL]	Airflow resistivity [Ns/m^4^]	Porosity [–]	Tortuosity [–]	Viscous characteristic length [μm]	Thermal characteristic length [μm]
1	0.0	1663	0.75	1.01	191	287
5	0.8	1001	0.65	1.02	184	245
6	1.6	1143	0.50	1.04	146	173
7	3.2	983	0.51	1.02	134	170

By means of these microstructural modifications, however, we can note that glycerol influences only high frequencies and that above 1.6 mL of glycerol (sample 6) there is a minimal variation, even if the quantity is doubled (sample 7).

Table 7 reports the average sound absorption coefficients for the various samples. This reveals a noteworthy increase in sound absorption coefficient (side A) when glycerol is added to the alginate-based sample. This improvement can be quantified as 9% for the first step (0–0.8 ml), and a further 5–6% when twice as much glycerol is added.

**Table 7:** Averaged sound absorption coefficient with increasing glycerol concentration.

Material	Glycerol [mL]	α_w_ – side A	α_w_ – side B
1	0.0	0.37	0.59
5	0.8	0.46	0.69
6	1.6	0.51	0.66
7	3.2	0.50	0.65

As regards side B, our investigation shows that adding glycerol improves sound absorption at the peak frequency; when adding 1.6 or 3.2 mL of glycerol (samples 6 and 7 respectively), the peak moves to higher frequencies, with no significant differences between 1.6 and 3.2. Here, as when adding alginate, the resistive layer generated by the microplastics yields different sound absorption performance values as compared to side A. Consequently, there is no need to study the microstructure using the JCA model. It is, however, important to note that adding glycerol enhances the averaged sound absorption coefficient of 10% (Table 7), but further addition does not lead to further important improvements.

Overall, we can conclude that glycerol addition improved acoustic absorption at most of the tested concentrations, considering noise exposure to side A. However, once a certain amount of glycerol is added, no more performance enhancement is obtained (sample 7). In contrast, a small quantity of glycerol does improve the acoustic absorption of side B (resistive layer), especially at middle–high frequencies, but when the glycerol concentration is further increased, the performance of this side starts to deteriorate again.

#### Powder type variation

**Figure 10:**
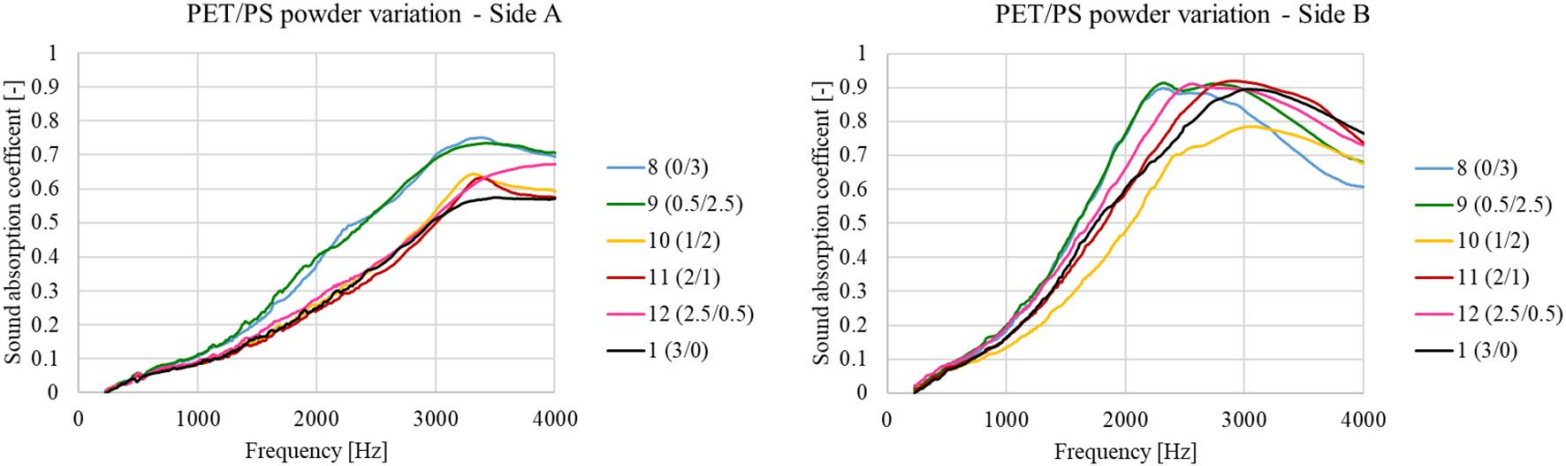
Variation of sound absorption frequency coefficient with varying microplastics powder ratios.

Varying the type of microplastics powder (PET and PS) within a fixed 1.4 w/v % alginate matrix produces differences in sound absorption. Figure 10 reports the measured frequency trends for both side A and side B.

Focusing on side A, it is interesting to note that slightly increasing the PET/PS ratio (from 0.0/3.0—sample 8, to 0.5/2.5—sample 9) has no effect on sound absorption performance. However, further increases in PET/PS (1.0/2.0—sample 10, and 2.0/1.0—sample 11) lead to reductions in sound absorption performance and absorption focusing at about 3000 Hz. As seen in Fig. 10 (left), at higher PET/PES ratios (3.0/0.0, Sample 1), the performance is worse still.

Table 8 reports the JCA parameters calculated by means of a minimization procedure. This showed that the PET/PS ratio has no substantial influence on tortuosity and only a small influence on porosity, when a small amount of PET is added during foam production. Conversely, PET content has an important influence on characteristic lengths. Specifically, the thermal characteristic length indicates very large cells in almost all cases, except in the samples with lower PET powder content (9 and 10). Thermal characteristic length was always higher than in sample 1, while viscous characteristic lengths present a random variation as well as flow resistivity (Table 8), which could be caused by the differing powder content in the matrix^51^. In fact, the PS powder tends to lay within the connections between cells, since it is lighter than the PET powder. It is interesting to highlight that, comparing sample 1 versus sample 12, a small insertion of PS (0.5 g) changes meaningfully the JCA parameters. This is due to the fact that during the production, PS presents a different behaviour than PET. PS tends to gather within the microstructure, while, as explained, PET accumulates on the surface. For this reason, even a small amount of PS is able to change the cell formation and thus the acoustic behaviour.

**Table 8:** Microplastics powder ratio variation affects JCA model parameters.

Sample	PET powder [g]	PS powder[ g]	Airflow resistivity [Ns/m^4^]	Porosity [–]	Tortuosity [–]	Viscous characteristic length [μm]	Thermal characteristic length [μm]
8	0.0	3.0	5668	0.91	1.56	165	721
9	0.5	2.5	2344	0.87	1.64	112	340
10	1.0	2.0	2606	0.85	1.61	102	302
11	2.0	1.0	7003	0.92	1.67	227	667
12	2.5	0.5	3305	0.94	1.69	151	947
1	3.0	0.0	1663	0.75	1.01	191	287

In any case, the cell shape in all samples was clearly characterized by large cavities joined by relatively small connections. Table 9 shows the average sound absorption coefficients, revealing that, once again, PET microplastics powder can increase sound absorption performance only when a small amount is added. When greater quantities are added, PET microplastics powder negatively affects final results.

**Table 9:** Variations in averaged sound absorption coefficient with alterations in microplastics powder composition.

Sample	PET powder [g]	PS powder [g]	α_w_—side A	α_w_—side B
8	0.0	3.0	0.47	0.60
9	0.5	2.5	0.58	0.64
10	1.0	2.0	0.44	0.52
11	2.0	1.0	0.47	0.60
12	2.5	0.5	0.49	0.62
1	3.0	0.0	0.37	0.59

Different behaviour was observed for the B side of samples. Specifically, the peak shifted from 3000 Hz (seen for side A) to about 2000 Hz. This shift is mainly due to the resistive layer created by microplastics aggregation in this part of the material. When the PET/PS ratio was altered from 0/3 to 0.5/2.5 (samples 8 and 9 respectively), an improvement in performance at high frequencies was observed, with a minimum for the ratio PET/PS = 1/2. However, if the ratio of PET to PS is further increased, we see the same behaviour as in side A, namely a sharp worsening of performance and a shift in the peak to about 3000 Hz. When increasing the ratio of PET to PS even further, however, the performance gets better, until it reaches a maximum after which no further improvement can be obtained. This could be caused by the resistive surface layer reaching its maximum acoustic efficiency. This is also confirmed by the averaged sound absorption coefficients, reported in Table 7.

#### Addition of PET powder and flakes

**Figure 11:**
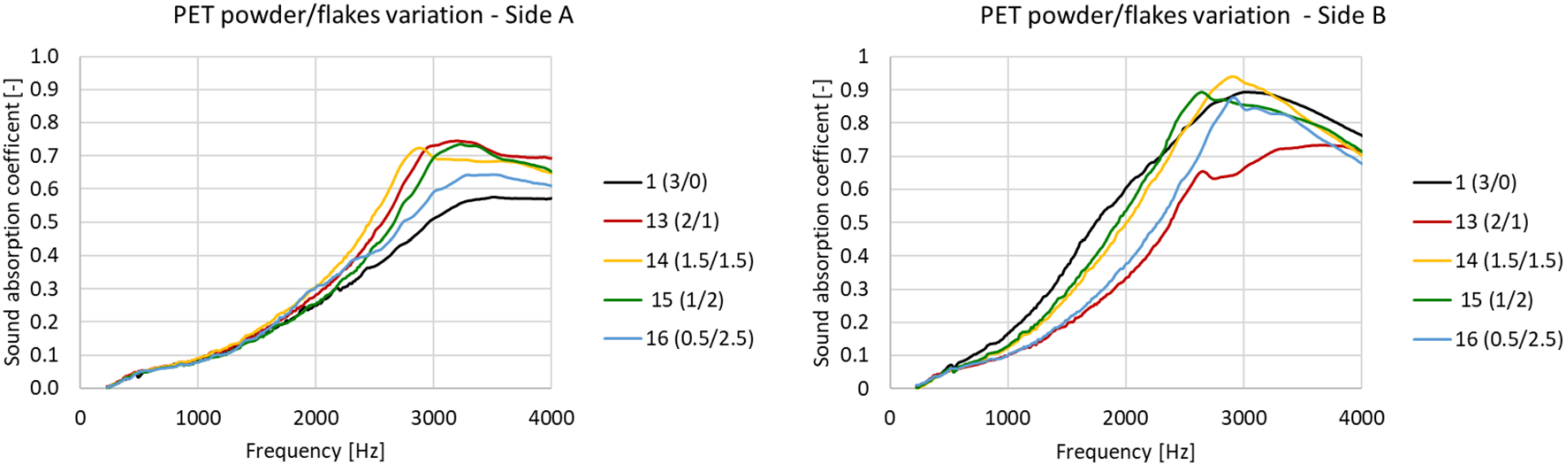
Variation in sound absorption coefficient frequency upon variation in microplastics powder/flakes ratio.

Figure 11 illustrates the sound absorption coefficients measured at different ratios of PET powder/flakes. This shows the influence of the different size/shapes of microplastics with respect to powder alone (see Fig. 10).

It is evident how the addition of flakes improves performance with respect to ground powder, at least up to a powder to flakes ratio 1/2. Referring to side A, better sound absorption is seen at a powder/flakes ratio of 2/1 (sample 13) than at 3/0 powder/flakes ratio (sample 1), especially at the peak frequency (about 3000 Hz).

As above, the JCA model was used to elucidate the main variation in the microstructure. As reported in Table 10, among the 5 parameters, it was the airflow resistivity that significantly increased. This indicates that the flakes modify the shape of the cells and that the connections between them are proportionally reduced. This is confirmed by the thermal characteristic length values; while in sample 1 (powder/flakes ratio = 3/0) the ratio between the two lengths is 0.66, in sample 13 (powder/flakes ratio = 2/1) the ratio is 0.42, which is 37% less than sample 1. Furthermore, cell dimension is significantly increased (2.3 times), the porosity of sample 13 being 12% greater (0.85).

**Table 10:** JCA model parameters for different combinations of microplastics.

Sample	PET powder [g]	PET flakes [g]	Airflow resistivity [Ns/m^4^]	Porosity [–]	Tortuosity [–]	Viscous characteristic length [μm]	Thermal characteristic length [μm]
1	3.0	0.0	1663	0.75	1.01	191	287
13	2.0	1.0	3721	0.85	1.71	290	680
14	1.5	1.5	4007	0.81	1.51	234	468
15	1.0	2.0	4893	0.79	1.98	128	443
16	0.5	2.5	6691	0.81	1.97	333	698

The variation in tortuosity is a particularly interesting finding; this parameter indicates the free mean wave path inside the material thickness. In sample 13, the tortuosity is almost doubled in comparison to sample 1, meaning that the flakes in the matrix have a noteworthy effect, modifying the microstructure of the foam. Although PET tendentially sinks to side B, it is evident that not all the flakes reach the bottom and the few remaining suspended are sufficient to provoke these important changes in the end product.

At a powder/flakes ratio of 1.5/1.5 (sample 14), things changed slightly. Airflow resistivity increased, while porosity, tortuosity and thermal lengths did not. It is therefore possible to conclude that raising the flakes content provokes a modification in the foam’s microstructure and, as a consequence, its overall sound absorption. In this case, a performance reduction limited to the peak frequency is observed. This tendency was confirmed by sample 15 (powder/flakes ratio = 1/2), where PET flakes became the principal microplastic component. In comparison to sample 14, the modification is significant and consequently affects airflow resistivity (see Table 10).

While tortuosity presented noteworthy variations, porosity, on the other hand, did not. This implies that the flakes tended to accumulate between cells, impairing the ability of the connections to transmit waves within the material (fluid phase). The decreasing viscous characteristic length confirms this hypothesis and indicates that very few other void spaces are available in the matrix for elongated powder granulates (flakes). Accordingly, when reaching the greatest proportion of flakes (sample 16, powder/flakes ratio = 0.5/2.5), airflow resistivity rises considerably, while tortuosity and porosity do not.

Thermal length values highlight a significant cell variation, showing that the sample contains large cells. The overall sound absorption decreases mainly at the peak frequency, while (Table 11) no significant differences were found in the mid–high frequencies range. Acoustic absorption is improved by adding PET flakes (samples 13 and 14), but when flakes start to become the foremost component of the PET filler, the overall performance diminishes back to almost the starting point.

**Table 11:** Averaged sound absorption coefficient for different combinations of microplastic powder and flakes.

Sample	PET powder [g]	PET flakes [g]	α_w_—side A	α_w_—side B
1	3.0	0.0	0.37	0.59
13	2.0	1.0	0.44	0.46
14	1.5	1.5	0.43	0.55
15	1.0	2.0	0.41	0.57
16	0.5	2.5	0.40	0.49

Again, side B demonstrated different behaviour with respect to side A, due to the resistive layer. The sound absorption coefficient increased to about 3000 Hz as flakes were added and then started to fall off. Increasing the proportion of microplastic flakes only slightly modifies the curves, but when the lowest flakes content is added (sample 13, powder/flakes ratio = 2/1), the performance sharply decreases. This is caused by a modification in the superficial layering, which fails to act as an efficient resistive layer. This could be due to one or more of the following:

- the flake distribution is patchy, with random areas of presence or absence, rather than forming a continuous restive layer;
- small amounts of flakes prevent the formation of a homogeneous resistive layer in side B, where the PET sinks to;
- flakes get stuck in the connections between cells, preventing PET powder from homogeneously sinking to side B;
- a combination of the options above reported.

**Figure 12:**
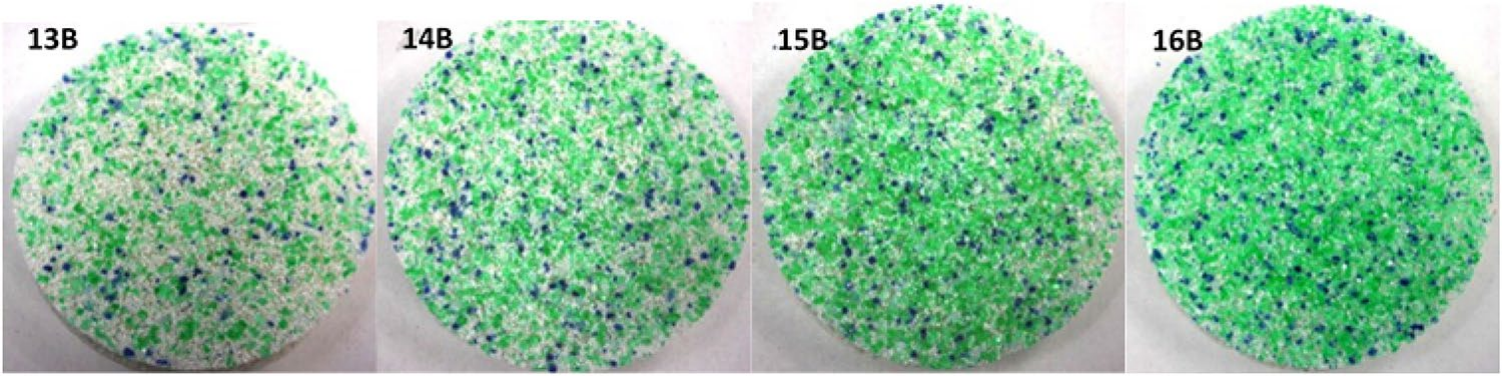
Pictures of side B of samples 13 to 16.

In the other cases, the addition of a larger amount of PET flakes caused the sound absorption performance to improve. As shown in Fig. 9, the greater presence of elongated granules led them to sink to side B, thus providing the resistive layer effect. Accordingly, sample 14 (powder/flakes ratio = 1.5/1.5) displayed almost the same properties as sample 1, while sample 15 (powder/flakes ratio = 1/2), exhibited a decrease in sound absorption performance. This trend was confirmed by sample 16 (powder/flakes ratio = 0.5/2.5) where, in the frequency range below the peak, sound performance was reduced. Figure 12 shows the B side of samples 13 to 16; the increasing surface density as the amount of flakes increased is clearly visible.

These observations are also confirmed by the averaged values for the sound absorption coefficient; as shown in Table 11, performance significantly decreased in comparison to sample 1 (no PET flakes) when microplastic flakes were added. In samples 14 (powder/flakes ratio = 1.5/1.5) and 15 (powder/flakes ratio = 1/2) sound absorption improved, but again worsened for sample 16 (greatest quantity of flakes, powder/flakes ratio = 0.5/2.5) to below the initial improvement.

Overall, it can be concluded that granule size/shape (addition of flakes) can slightly improve acoustic absorption at side A when a small amount is added to the foam. Conversely, addition of flakes never improves the performance of side B (resistive layer).

### Comparison with available tuneable materials

Several works related to acoustic tuneable materials are present in the literature. Most of them are related to metamaterials and phononic crystals. Chen et al.^52^ presented a complete review on tuneable acoustic materials, focusing on metamaterial properties. Outcomes clearly highlight that narrow working bands are provided by these solutions because of their local resonant behaviour, thus preventing most of the practical implementation. Other similar technologies can be applied. For example, Wu et al.^53^ designed and tested an aluminium plate for low-frequency tuneable sound absorbing system, using split tube resonators. This system involves a complex structure providing very good sound absorption but in a very narrow frequency band. Tuning the properties of materials using voltage was also explored before^54^, again providing narrow-frequency properties of the end product. When focusing on microstructure, Kanoshi et al.^55^ provided a very good work concerning a micro-hole array for acoustic purposes. Even if the system is newly constructed, the acoustic behaviour is comparable to the one proposed in this work. This demonstrates how the used microplastics change the acoustic properties of the open-cell foam by modifying the microstructure of the holes and thus tuning its final acoustic performances. Rare are the examples of acoustic open-cell tuneable foam in the literature. Xu et al.^13^ obtained different acoustic characteristics of the same epoxy foams by means of expandable microspheres. Some tuneable results are obtained but at the same frequency for 3 samples out of five while for the other two very poor performances are verified. Interestingly, Rapisarda et al.^56^ produced an ultralight aerogel with tuneable acoustic properties, similar to those here presented. In conclusion, no other waste recycling experience is included in the literature focusing on bio-based open-cell foam production, featuring customization of the acoustic properties by adding microplastics. The obtained results are in line with the ones of other end products, with the addition of a very important recycling aspect.

## Conclusions

This paper presents a customized clean method for producing open-cell foams with tuneable sound absorption characteristics. The tailoring of the properties can be performed varying microplastics type and content in a bio-based matrix. In this way, a new sustainable and clean solution to marine litter recycling is proposed, tested and validated. Various types of plastics were included in the samples, with different shapes, kinds and colours. Results clearly indicate that it is possible to produce sustainable open-cell foams for acoustics applications. The main findings can be summarized as follows:

- the foams produced provide very good sound absorption performances in most cases;
- the sound absorption performance of these materials is tuneable, able to provide different sound absorption frequency peaks, depending on the type and shape of microplastics included;
- microplastic types (PET or PS) and shapes (powder or flakes) distribute differently in the foam structure due to intentional accumulation of microplastics on one side of the foam;
- this accumulation of microplastics on one side of the samples provides a desirable acoustic-resistive layer, capable of enhancing the acoustic properties of all samples;
- the microstructure of the material can be modified by varying the microplastic powder and flakes composition, causing noteworthy differences in the 5 Johnson–Champoux–Allard acoustic parameters;
- increasing the alginate concentration in the matrix increases the samples’ performance and stiffness, with substantial differences between the side where the microplastics are deposited (acoustic-resistant layer) and the other one;
- the plasticizer also acts to enhance performance on both sides and, remarkably, frequency peak shifts are possible in the resistive layer side;
- the addition of PS to mixed microplastic powder compositions (PS and PET) results in improved sound absorption by the open porous structure facing the sound waves. When switching to the other side, a peak frequency shift is observed, suggesting it is possible to tune overall performance;
- the shape of the microplastics influences the final outcome. Specifically, sound absorption can be improved by adding a small amount of microplastic flakes instead of powder when no resistive layer is opposed to the sound waves. In all the other cases, microplastic flakes exert a negative influence on the overall performance.

As a whole, this research demonstrates how it is possible to recycle marine microplastics litter to produce new clean materials able to serve as acoustic absorption layers for civil, industrial and naval applications.

## Data Availability

All data generated or analysed during this study are included in this published article.

## References

[1] Bolton JS, Kang YJ (1997). Elastic porous materials for sound absorption and transmission control. SAE Trans..

[2] Matsuda M, Nomura T, Iwai H (1998). Material consolidation for automotive interior and exterior parts through development of a high performance material. SAE Tech. Pap..

[3] Park UH, Heo JH, Lee IS, Park DK (2020). The process development for sound absorption design of non-woven car mat. SSP.

[4] Sung G, Kim JW, Kim JH (2016). Fabrication of polyurethane composite foams with magnesium hydroxide filler for improved sound absorption. J. Ind. Eng. Chem..

[5] Davy JL (2010). The improvement of a simple theoretical model for the prediction of the sound insulation of double leaf walls. J. Acoust. Soc. Am..

[6] Zamora Mestre JL, Niampira A (2020). Lightweight ventilated façade: Acoustic performance in laboratory conditions, analysing the impact of controlled ventilation variations on airborne sound insulation. Build. Acoust..

[7] Yu X, Lau S-K, Cheng L, Cui F (2017). A numerical investigation on the sound insulation of ventilation windows. Appl. Acoust..

[8] Mehta M, Johnson J, Rocafort J (1999). Architectural Acoustic: Principles and Design.

[9] Lee J, Kim G-H, Ha C-S (2012). Sound absorption properties of polyurethane/nano-silica nanocomposite foams. J. Appl. Polym. Sci..

[10] Kim S-R, Kim H-S, Kim J-S, Kim B-K, Lee S-H (2012). Sound insulation performance of prefabricated cabins in cruise ships. Noise Control Eng. J..

[11] Seiler RD, Holbach G (2013). Acoustic quality on board ships. Proc. Acoust..

[12] Yankaskas K, Fischer R, Spence J, Komrower J (2017). Engineering out the noise. Hear. Res..

[13] Xu Y, Li Y, Zhang A, Bao J (2017). Epoxy foams with tunable acoustic absorption behavior. Mater. Lett..

[14] Zhang H, Xiao Y, Wen J, Yu D, Wen X (2016). Ultra-thin smart acoustic metasurface for low-frequency sound insulation. Appl. Phys. Lett..

[15] Asdrubali F, D’Alessandro F, Schiavoni S (2015). A review of unconventional sustainable building insulation materials. Sustain. Mater. Technol..

[16] Densley Tingley D, Hathway A, Davison B (2015). An environmental impact comparison of external wall insulation types. Build. Environ..

[17] Ingrao C (2014). Recycled-PET fibre based panels for building thermal insulation: Environmental impact and improvement potential assessment for a greener production. Sci. Total Environ..

[18] Cozzarini L, Marsich L, Ferluga A, Schmid C (2020). Life cycle analysis of a novel thermal insulator obtained from recycled glass waste. Dev. Built Environ..

[19] Schmid C, Cozzarini L, Zambello E (2021). Microplastic’s story. Mar. Pollut. Bull..

[20] Ajith N, Arumugam S, Parthasarathy S, Manupoori S, Janakiraman S (2020). Global distribution of microplastics and its impact on marine environment: A review. Environ. Sci. Pollut. Res..

[21] Andrady AL (2017). The plastic in microplastics: A review. Mar. Pollut. Bull..

[22] Halim Hamid S (2020). Handbook of Polymer Degradation.

[23] O’Brine T, Thompson RC (2010). Degradation of plastic carrier bags in the marine environment. Mar. Pollut. Bull..

[24] Dijkgraaf E, Vollebergh H (2004). Burn or bury? A social cost comparison of waste disposal methods. Ecol. Econ..

[25] Franke H-J (1999). Improvement of carbon burn-up during fluidized bed incineration of plastic by using porous bed materials. Energy Fuels.

[26] Ahmetli G, Kocaman S, Ozaytekin I, Bozkurt P (2013). Epoxy composites based on inexpensive char filler obtained from plastic waste and natural resources. Polym. Compos..

[27] Chandni TJ, Anand KB (2018). Utilization of recycled waste as filler in foam concrete. J. Build. Eng..

[28] Catanzano O (2018). Macroporous alginate foams crosslinked with strontium for bone tissue engineering. Carbohydr. Polym..

[29] Grant GT, Morris ER, Rees DA, Smith PJC, Thom D (1973). Biological interactions between polysaccharides and divalent cations: The egg-box model. FEBS Lett..

[30] Porrelli D (2015). Alginate-hydroxyapatite bone scaffolds with isotropic or anisotropic pore structure: Material properties and biological behavior. Macromol. Mater. Eng..

[31] Turco G (2009). Alginate/Hydroxyapatite biocomposite for bone ingrowth: A trabecular structure with high and isotropic connectivity. Biomacromolecules.

[32] Caniato, M. & Travan, A. *Method for Recycling Waste Material* (2019).

[33] Gao C, Pollet E, Avérous L (2017). Properties of glycerol-plasticized alginate films obtained by thermo-mechanical mixing. Food Hydrocolloids.

[34] Olivas GI, Barbosa-Cánovas GV (2008). Alginate–calcium films: Water vapor permeability and mechanical properties as affected by plasticizer and relative humidity. LWT Food Sci. Technol..

[35] Travan A (2016). Hyaluronan delivery by polymer demixing in polysaccharide-based hydrogels and membranes for biomedical applications. Carbohydr. Polym..

[36] Arthur, C., Baker, J. & Bamford, H. Proceedings of the international research workshop on the occurrence, effects and fate of microplastic marine debris. In *NOAA Technical Memorandum NOS-OR&R-30* (2009).

[37] Erni-Cassola G, Zadjelovic V, Gibson MI, Christie-Oleza JA (2019). Distribution of plastic polymer types in the marine environment. A meta-analysis. J. Hazard. Mater..

[38] Gago J, Galgani F, Maes T, Thompson RC (2016). Microplastics in seawater: Recommendations from the marine strategy framework directive implementation process. Front. Mar. Sci..

[39] Zhang YS, Khademhosseini A (2017). Advances in engineering hydrogels. Science.

[40] International Organization for Standardization. *10534–2:1998 Acoustics—Determination of Sound Absorption Coefficient and Impedance in Impedance Tubes—Part 2: Transfer-Function Method*. (1998).

[41] Atiénzar-Navarro R, del Rey R, Alba J, Sánchez-Morcillo VJ, Picó R (2020). Sound absorption properties of perforated recycled polyurethane foams reinforced with woven fabric. Polymers.

[42] Champoux Y, Allard J (1991). Dynamic tortuosity and bulk modulus in air-saturated porous media. J. Appl. Phys..

[43] Allard J, Atalla N (2009). Propagation of Sound in Porous Media: Modelling Sound Absorbing Materials.

[44] Lagarias JC, Reeds JA, Wright MH, Wright PE (1998). Convergence properties of the Nelder-Mead simplex method in low dimensions. SIAM J. Optim..

[45] Deshmukh S, Ronge H, Ramamoorthy S (2019). Design of periodic foam structures for acoustic applications: Concept, parametric study and experimental validation. Mater. Des..

[46] Mosanenzadeh SG, Doutres O, Naguib HE, Park CB, Atalla N (2015). A numerical scheme for investigating the effect of bimodal structure on acoustic behavior of polylactide foams. Appl. Acoust..

[47] Yang XH (2015). A simplistic unit cell model for sound absorption of cellular foams with fully/semi-open cells. Compos. Sci. Technol..

[48] Ji Y, Chen S, Zhu W (2020). The effect of pore numbers in the cell walls of soybean oil polyurethane foam on sound absorption performance. Appl. Acoust..

[49] Bonfiglio P, Pompoli F (2013). Inversion problems for determining physical parameters of porous materials: Overview and comparison between different methods. Acta Acust. Acust..

[50] International Organization for Standardization. *ISO 9053–2:2020 Acoustics—Determination of airflow resistance—Part 2: Alternating airflow method*.

[51] González-Rivera J, Iglio R, Barillaro G, Duce C, Tinè MR (2018). Structural and thermoanalytical characterization of 3D porous PDMS foam materials: The effect of impurities derived from a sugar templating process. Polymers.

[52] Chen S (2018). A review of tunable acoustic metamaterials. Appl. Sci..

[53] Wu X (2016). Low-frequency tunable acoustic absorber based on split tube resonators. Appl. Phys. Lett..

[54] Xiao S, Tang ST, Yang Z (2017). Voltage-tunable acoustic metasheet with highly asymmetric surfaces. Appl. Phys. Lett..

[55] Konishi S, Yoda M, Sugiyama S, Akishita S (2000). Tunable acoustic absorber using a micro acoustic hole array. Electron. Comm. Jpn..

[56] Rapisarda M, Malfense Fierro G-P, Meo M (2021). Ultralight graphene oxide/polyvinyl alcohol aerogel for broadband and tuneable acoustic properties. Sci. Rep..

